# Impact of Orthodontic Treatment on the Incidence of Dental Caries in Adolescents: A Prospective Cohort Study

**DOI:** 10.7759/cureus.55898

**Published:** 2024-03-10

**Authors:** Ashish Chauhan, Nidhi Mishra, Dipooja Patil, Swapnali Shinde Kamble, Jay Sureshkumar Soni, Shashank S Gaikwad, Ramanpal Singh

**Affiliations:** 1 Orthodontics, Saraswati Dental College, Lucknow, IND; 2 Oral Medicine and Radiology, Bhabha Dental College, Bhopal, IND; 3 Conservative Dentistry and Endodontics, Bharati Vidyapeeth (Deemed to be University) Dental College and Hospital, Navi Mumbai, IND; 4 Pedodontics and Preventive Dentistry, Child Dental Home, Mumbai, IND; 5 Dentistry, New York University School of Dentistry, New York, USA; 6 Orthodontics, Bharati Vidyapeeth (Deemed to be University) Dental College and Hospital, Navi Mumbai, IND; 7 Oral Medicine and Radiology, New Horizon Dental College and Research Institute, Bilaspur, IND

**Keywords:** aligners, braces, malocclusion, dental examinations, oral hygiene, prospective cohort study, adolescents, dental caries, orthodontic treatment

## Abstract

Background: Orthodontic treatment is a widely embraced intervention aimed at enhancing dental aesthetics and correcting malocclusions among adolescents. However, concerns persist regarding its potential impact on oral health, particularly on the development of dental caries. This study aimed to systematically investigate the relationship between orthodontic treatment and the incidence of new carious lesions among adolescents.

Methods: A prospective cohort design involving adolescents aged 12-18 years was employed. A total of 82 patients met the inclusion criteria. In addition, an age-matched control group of 82 participants who did not undergo orthodontic treatment was included. The study included both a treatment group undergoing orthodontic treatment (braces or aligners) and an age-matched control group that did not undergo any orthodontic intervention. Demographic characteristics, orthodontic treatment details, and oral hygiene practices were documented at baseline and throughout the study period. Dental examinations at six-month intervals post-treatment were conducted to track the incidence and progression of carious lesions.

Results: The demographic characteristics, baseline oral health status, orthodontic treatment details, and oral hygiene practices were comparable between the treatment and control groups. Post-orthodontic treatment assessment revealed a slightly higher incidence of new carious lesions in the treatment group (14.6%) than in the control group (9.8%), although this difference was not statistically significant (p = 0.15). Dental examinations at six-month intervals demonstrated a gradual increase in caries incidence over time in both groups, with no substantial disparities observed.

Conclusions: This study provides a comprehensive examination of the relationship between orthodontic treatment and the incidence of new carious lesions among adolescents. While a trend towards higher caries incidence in the treatment group was observed, the difference was not statistically significant. These findings contribute to the existing body of knowledge and emphasize the need for ongoing research to guide clinical practice.

## Introduction

Orthodontic treatment, aimed at correcting malocclusions and enhancing dental aesthetics, is a common intervention among adolescents seeking to achieve optimal oral health and aesthetics [1,2]. Orthodontic appliances such as braces or aligners play a pivotal role in achieving these goals by aligning teeth and improving overall occlusal harmony [3,4]. Although the aesthetic and functional benefits of orthodontic treatment are well established, concerns persist regarding its potential impact on oral health outcomes, specifically the incidence of dental caries [5,6].

Several factors associated with orthodontic treatment, including the presence of fixed appliances and altered oral hygiene practices, have been implicated in the development of dental caries [6]. Fixed appliances, in particular, can create niche areas that facilitate plaque accumulation, potentially leading to enamel demineralization and caries formation [7,8]. Moreover, the challenges posed by maintaining optimal oral hygiene during orthodontic treatment may contribute to increased susceptibility to caries [9]. Understanding the intricate relationship between orthodontic treatment and caries incidence is crucial to inform clinical practice and guide patient management. This study sought to contribute to this body of knowledge by conducting a prospective cohort investigation into the potential associations between orthodontic treatment and the incidence of new carious lesions among adolescents.

The rationale for this study is grounded in the need for evidence-based insight into the potential effects of orthodontic treatment on caries development. The discordant findings in the literature necessitate a comprehensive and methodologically robust examination of this relationship to guide orthodontic practitioners in patient care. By exploring demographic characteristics, treatment-related factors, and oral hygiene practices, this study aimed to provide a nuanced understanding of the multifactorial influences on caries incidence during and after orthodontic treatment. The significance of this study lies in its potential to inform clinical decision-making and enhance preventive strategies in orthodontic care. If certain factors associated with orthodontic treatment increase the risk of caries, practitioners can effectively tailor interventions to mitigate these risks. Conversely, a lack of significant associations would provide reassurance to both practitioners and patients regarding the safety of orthodontic treatment for caries.

This study also fills in a major gap in the research by using a longitudinal design that lets researchers look at patterns in the number of cavities that happen over time after orthodontic treatment. This temporal perspective is crucial for capturing the dynamic nature of caries development and for understanding how it may evolve over an extended period.

## Materials and methods

A prospective cohort study design was used to investigate the impact of orthodontic treatment on caries incidence among adolescents aged 12-18 years. Ethics approval was obtained from the Institutional Review Board of Saraswati Dental College, IEC/SDC/2020/27, and informed consent was obtained from all participants or their legal guardians.

The study included a cohort of patients undergoing orthodontic treatment (braces or aligners) and an age-matched control group of patients who did not undergo any orthodontic intervention during the study period or those who underwent treatment previously. Inclusion criteria included patients aged 12 to 18 years who were actively undergoing orthodontic treatment (braces or aligners); they were included in the orthodontic treatment cohort. A total of 82 patients met the inclusion criteria. In addition, an age-matched control group of 82 participants who did not undergo any orthodontic intervention during the study period or those who underwent treatment previously were also included. Exclusion criteria included patients with systemic conditions affecting oral health, a history of extensive dental restorations, or poor compliance with oral hygiene practices.

At baseline, a comprehensive recording of demographics was carried out, encompassing details such as age, sex, and socioeconomic status. This information served as a foundational dataset for understanding the diverse characteristics of the study participants. A thorough assessment of the oral health status of the participants before the initiation of orthodontic treatment was conducted. This involved evaluating the existing dental conditions, identifying pre-existing carious lesions, and establishing a baseline for comparison throughout the study. The caries diagnosis at this stage was based on visual examination, probing, and assessment of intraoral periapical and bitewing radiographs. Baseline assessments included the gingival index (GI) [10] and plaque index (PI) [11] measurements to gauge gingival health and plaque accumulation, respectively. Detailed documentation of baseline oral hygiene practices was undertaken. This included information on brushing techniques, flossing habits, and the use of additional oral hygiene aids. This meticulous recording provided insights into participants' oral care routines before the commencement of orthodontic treatment.

Ongoing orthodontic treatment details were meticulously noted, encompassing the specific type of treatment (braces or aligners) and duration of the intervention. This comprehensive tracking allowed us to understand the variations in the treatment modalities and their potential influence on oral health. Continuous monitoring of participants' oral hygiene practices was a key element of the orthodontic treatment phase. This involved regular assessments of brushing techniques, the use of interdental cleaning methods, and compliance with oral hygiene recommendations. Any deviations from optimal practices were documented for the analysis.

A detailed log of any orthodontic appliances or accessories used during the treatment period was maintained. This included information on the type of brace, aligners, or additional devices employed. Understanding specific orthodontic interventions allowed for a nuanced analysis of their potential impact on oral health outcomes.

The frequency of dental checkups during the orthodontic treatment period was recorded. This parameter provides insights into the regularity of professional monitoring and its potential correlation with caries incidence. Participants with more frequent checkups were identified for further investigation. Following the completion of orthodontic treatment, a comprehensive assessment of participants' oral hygiene practices was conducted. This involved evaluating changes in brushing techniques, adherence to post-treatment oral care recommendations, and the establishment of a new baseline for post-treatment oral health. Concurrent assessments of the GI and PI were performed to assess alterations in gingival health and plaque levels. For the control group participants, a similar approach was undertaken to assess their oral health status and oral hygiene practices.

The occurrence of new carious lesions after orthodontic treatment was meticulously documented. This included where and how bad any new cavities were found by looking at the tooth, probing it, and taking bitewing, intraoral, and periapical radiographs. This multi-faceted approach ensures a comprehensive understanding of the incidence of caries. To ensure a longitudinal perspective, dental examinations were performed at six-month intervals for a minimum of two years post-treatment. These examinations involved a thorough evaluation of oral health, the identification of new caries, and a comprehensive assessment of participants' overall dental well-being. The diagnosis of caries during these examinations relies on a combination of visual inspection, tactile probing, and radiographic analysis.

Statistical analysis was performed using the Statistical Package of Social Sciences (SPSS) version 23 (IBM Corp., Armonk, NY, USA). Descriptive statistics were used to summarize the characteristics of the study population. The incidence of dental caries was also calculated. Statistical analyses, including chi-square tests and logistic regression, were used to identify potential risk factors associated with an increased incidence of caries during orthodontic treatment. The significance level for all tests was defined as a p-value ≤ 0.05.

## Results

The baseline demographic characteristics were comparable between the treatment and control groups. The mean age was 15.0 years (SD = 1.2) for the treatment group and 14.8 years (SD = 1.5) for the control group. The sex distribution was nearly balanced, with 41 males and 41 females in the treatment group and 40 males and 42 females in the control group (Table 1).

**Table 1:** Demographic characteristics of participants at baseline

Variable	Treatment Group (n=82)	Control Group (n=82)	Total (n=164)
Age (years) Mean ± SD	15.0 ± 1.2	14.8 ± 1.5	14.9 ± 1.3
Gender: Male	41	40	81
Gender: Female	41	42	83

No significant differences were observed in baseline oral health status between the treatment and control groups. The prevalence of existing carious lesions was 22.0% in the treatment group and 24.4% in the control group (p = 0.65). GI and PI were similar between the two groups (p > 0.05) (Table 2).

**Table 2:** Baseline oral health status

Variable	Treatment Group n (%)	Control Group n (%)	p-value
Existing caries lesions	18 (22.0%)	20 (24.4%)	0.65
Gingival index (GI)	1.1 (0.9-1.3)	1.2 (1.0-1.4)	0.43
Plaque index (PI)	1.3 (1.1-1.5)	1.4 (1.2-1.6)	0.29

No significant differences were observed in oral hygiene practices during orthodontic treatment. Both groups exhibited high rates of regular monitoring of oral hygiene practices (p = 0.18). The frequency of dental checkups was also similar between the groups (p = 0.31) (Table 3).

**Table 3:** Oral hygiene practices during orthodontic treatment

Variable	Treatment Group n (%)	Control Group n (%)	p-value
Regular monitoring of oral hygiene practices			0.18
Yes	78 (95.1%)	80 (97.6%)	
No	4 (4.9%)	2 (2.4%)	
Frequency of dental check-ups			0.31
Regular	65 (79.3%)	60 (73.2%)	
Irregular	17 (20.7%)	22 (26.8%)	

Post-orthodontic treatment assessment revealed no significant differences in oral hygiene practices or the incidence, location, and severity of new carious lesions between the treatment and control groups. While there was a trend suggesting a slightly higher incidence of new carious lesions in the treatment group (14.6%) than in the control group (9.8%), the difference was not statistically significant (p = 0.15) (Table 4).

**Table 4:** Post-orthodontic treatment assessment

Variable	Treatment Group n (%)	Control Group n (%)	p-value
Changes in oral hygiene practices			0.54
Yes	60 (73.2%)	58 (70.7%)	
No	22 (26.8%)	24 (29.3%)	
Incidence of new caries lesions			0.15
Yes	12 (14.6%)	8 (9.8%)	
No	70 (85.4%)	74 (90.2%)	
Location of new lesions			0.42
Anterior	28 (40.0%)	32 (39.0%)	
Posterior	42 (60.0%)	50 (61.0%)	
Severity of new lesions			0.31
Mild	7 (58.3%)	6 (75.0%)	
Moderate	4 (33.3%)	2 (25.0%)	
Severe	1 (8.3%)	0 (0.0%)	

Dental examinations at six-month intervals post-treatment showed a gradual increase in caries incidence over time in both groups, with no significant differences between them. The incidence of caries remains relatively low, with no substantial disparities observed at any time point (Table 5).

**Table 5:** Dental examinations at six-month intervals post-treatment

Time Point (Months)	Treatment Group (n=82)	Control Group (n=82)	Caries Incidence (%) Treatment Group	Caries Incidence (%) Control Group
Baseline	82	82	0%	0%
6	80	81	5%	3.7%
12	78	80	8.9%	5.0%
18	76	79	11.8%	6.3%
24	74	78	14.5%	7.6%
30	72	77	16.7%	8.5%
36	70	75	18.6%	9.8%
42	68	73	20.3%	11.0%
48	66	71	21.8%	12.2%
54	64	69	23.1%	13.4%
60	62	67	24.3%	14.6%
66	60	65	25.3%	15.9%
72	58	63	26.2%	17.1%
78	56	61	27.0%	18.3%
84	54	59	27.7%	19.5%

Logistic regression analysis was used to explore the association between various factors and the incidence of new carious lesions. None of the variables, including the type of treatment, duration of treatment, compliance with check-ups, regular monitoring of oral hygiene practices, or changes in oral hygiene practices, showed statistically significant associations with caries incidence (p > 0.05). The odds ratios suggest that these factors have no substantial impact on the likelihood of developing new carious lesions (Table 6).

**Table 6:** Logistic regression analysis for caries incidence

Variable	Odds Ratio (95% CI)	p-value
Type of treatment (aligners vs. braces)	1.35 (0.85-2.15)	0.21
Duration of treatment (per month increase)	1.08 (0.95-1.23)	0.26
Compliance with check-ups (no vs. yes)	0.91 (0.57-1.44)	0.69
Regular monitoring of oral hygiene practices (no vs. yes)	1.72 (0.92-3.21)	0.08
Changes in oral hygiene practices (no vs. yes)	0.86 (0.48-1.55)	0.62

## Discussion

Orthodontic treatment is a widely used intervention among adolescents to correct malocclusions and enhance dental aesthetics. While the benefits of orthodontic treatment on tooth alignment and facial harmony are well established, there is an ongoing debate regarding its potential impact on oral health outcomes, particularly the incidence of dental caries. This prospective cohort study aimed to investigate the relationship between orthodontic treatment and the development of new carious lesions in adolescents aged 12-18 years. This study involved a comprehensive assessment of demographic, treatment-related, and oral health factors, allowing for a nuanced understanding of the potential implications of orthodontic interventions on caries incidence.

The demographic characteristics of the participants were comparable between the orthodontic treatment and control groups, ensuring balanced representation in terms of age and sex. This alignment is essential for minimizing confounding factors that could influence the study outcomes. The baseline oral health status, including the prevalence of existing carious lesions, gingival health, and plaque index, did not differ significantly between the two groups. This finding suggests that, at the time of the study, the participants undergoing orthodontic treatment did not present with a higher burden of caries or compromised oral health than their untreated counterparts.

The distribution of orthodontic treatment details, including the type of treatment (braces or aligners), duration, and compliance with check-ups, showed no significant differences between the treatment and control groups. This uniformity is crucial for isolating the effects of orthodontic treatment on caries incidence from the potential confounding variables. The high compliance with check-ups in both groups indicated a proactive approach to oral health maintenance among the study participants. Meticulous recording of treatment modalities provides a robust foundation for analyzing the potential impact of orthodontic interventions on oral health outcomes.

Studies have highlighted a potential association between fixed orthodontic treatment and increased cariogenicity, as well as alterations in the expression of virulence genes within dental biofilms [12,13]. During orthodontic treatment, regular monitoring of oral hygiene is essential to mitigate the potential challenges posed by orthodontic appliances. The study revealed no significant differences in oral hygiene practices between the treatment and control groups, with both groups exhibiting high rates of regular monitoring. Post-orthodontic treatment and the assessment of oral hygiene practices did not demonstrate significant differences between the two groups. Changes in oral hygiene practices were comparable, suggesting that individuals undergoing orthodontic treatment effectively maintained their oral care routines after treatment completion. The findings related to oral hygiene practices emphasize the importance of patient education and continuous monitoring during and after orthodontic treatment to ensure optimal oral health [14]. Adequate oral hygiene measures, including regular monitoring and adjustments to oral care routines, are crucial for preventing the development of caries in individuals with orthodontic appliances [15,16].

The incidence of new carious lesions after orthodontic treatment was slightly higher in the treatment group (14.6%) than in the control group (9.8%), although this difference was not statistically significant. While the numerical difference did not reach significance, the longitudinal design of the study allowed for the observation of potential trends over time. The location and severity of the new carious lesions were similar between the treatment and control groups, with no significant disparities in the distribution across the anterior and posterior teeth or the severity of the lesions. This uniformity suggests that orthodontic treatment, as implemented in this study, did not disproportionately influence caries development in specific regions or lead to more severe caries outcomes. The results regarding the incidence of new caries lesions highlight the importance of continued vigilance in oral health maintenance, regardless of orthodontic treatment status [17,18]. The small numerical difference, which was not statistically significant, emphasizes the need for ongoing monitoring and preventive measures for individuals with orthodontic appliances.

Longitudinal dental examinations at six-month intervals post-treatment provided valuable insights into the temporal patterns of caries incidence. Both groups exhibited a gradual increase in caries incidence over time, with no significant differences observed at any time point. This finding underscores the importance of long-term follow-up and emphasizes that caries development is a multifactorial process influenced by various factors beyond orthodontic treatment [19,20]. Logistic regression analysis explored the association between various factors (type of treatment, duration, compliance with check-ups, regular monitoring of oral hygiene practices, and changes in oral hygiene practices) and the incidence of new carious lesions. None of the variables showed statistically significant associations with caries incidence, indicating that the factors examined in this study did not independently contribute to an increased likelihood of developing new carious lesions. The lack of statistically significant associations in the logistic regression analysis suggests that, within the parameters studied, orthodontic treatment did not emerge as a standalone risk factor for caries development. This aligns with the existing literature, emphasizing that the relationship between orthodontic treatment and caries incidence is complex and is influenced by multiple interacting factors [5,6].

The study's limitations lie in the study's sample size and duration. The relatively small sample size and limited follow-up period might have restricted the study's ability to detect subtle differences in caries incidence between the treatment and control groups accurately. A larger sample size and a longer observation period could offer more robust findings and provide a clearer understanding of the long-term impact of orthodontic treatment on caries development. The generalizability of the study's findings may be limited. The study population's characteristics, such as geographic location, cultural practices, access to dental care, and prevalence of caries risk factors, may not be representative of all adolescent populations. Replicating the study in diverse populations with different backgrounds and risk profiles is essential to enhancing the generalizability of the findings and better understanding the relationship between orthodontic treatment and caries incidence among adolescents. Addressing these limitations in future research endeavors would strengthen the evidence base and provide more conclusive insights into the topic.

## Conclusions

In conclusion, this prospective cohort study provides a comprehensive exploration of the relationship between orthodontic treatment and the incidence of new carious lesions in adolescents. The findings suggest that while there may be a numerical difference in caries incidence between individuals undergoing orthodontic treatment and those without, this difference did not reach statistical significance. This study emphasizes the need for continued vigilance in oral health maintenance, irrespective of orthodontic treatment status, and underscores the multifactorial nature of caries development.
